# RF-Penetrable PET insert for simultaneous PET/MR imaging

**DOI:** 10.1186/2197-7364-1-S1-A5

**Published:** 2014-07-29

**Authors:** Brian J Lee, Alexander M Grant, Chen-Ming Chang, Craig S Levin

**Affiliations:** Department of Radiology, Stanford University, Stanford, CA 94305 USA; Molecular Imaging Program at Stanford (MIPS), Stanford, CA 94305 USA; Department of Physics, Stanford University, Stanford, CA 94305 USA; Department of Electrical Engineering, Stanford University, Stanford, CA 94305 USA; Department of Bioengineering, Stanford University, Stanford, CA 94305 USA; Department of Mechanical Engineering, Stanford University, Stanford, CA 94305 USA; Department of Applied Physics, Stanford University, Stanford, CA 94305 USA

Combined positron emission tomography (PET) and magnetic resonance imaging (MRI) shows promise to be a powerful tool for disease characterization as it enables the simultaneous measurement of molecular and anatomical information of the body. However, the impact of whole body simultaneous PET/MRI is limited by its high cost. To address this issue, we are developing an RF-penetrable PET insert that can be inserted into any MRI system without requiring modifications to the MR system hardware.

The proposed PET system prototype consists of 16 PET detector modules within Faraday cages in a 32 cm inner diameter ring pattern with small gaps between modules. These inter-modular gaps, along with electro-optical signal transmission technology that allows the PET insert to electrically float relative to the MRI RF ground, enable RF fields to pass through the PET ring with some attenuation.

In this study we investigate the feasibility of an RF-penetrable PET detector insert. We perform 2D (Maxwell, ANSOFT, USA) and 3D (XFdtd, REMCOM, USA) electromagnetic simulations as well as experiments in a 3T MR system (GE Healthcare, USA) to understand the degree to which a ring of electrically floating Faraday cages facilitates RF transmissivity. 2D electromagnetic simulation results show that a grounded PET insert blocks the RF field while an electrically floating PET insert allows the RF field to uniformly transmit through the gaps with some attenuation. The RF field attenuation levels of 3D simulation and experiments were 3.9 and 4.2~5.7 dB respectively, demonstrating that the simulation reasonably represents the experimental RF attenuation results.

We have shown from both simulations and experiments that the RF field of an MR system body coil can penetrate a PET ring through small inter-modular gaps, when the PET ring is electrically floating with respect to the MR system.

